# Exposure to lipophilic chemicals as a cause of neurological impairments, neurodevelopmental disorders and neurodegenerative diseases

**DOI:** 10.2478/intox-2013-0018

**Published:** 2013-09

**Authors:** Harold I. Zeliger

**Affiliations:** Zeliger Chemical, Toxicological, and Environmental Research, West Charlton, NY, USA

**Keywords:** neurological disease, Alzheimer's disease, Parkinson's disease, autism, ADHD, lipophilic chemicals, toxic chemicals

## Abstract

Many studies have associated environmental exposure to chemicals with neurological impairments (NIs) including neuropathies, cognitive, motor and sensory impairments; neurodevelopmental disorders (NDDs) including autism and attention deficit hyperactivity disorder (ADHD); neurodegenerative diseases (NDGs) including Alzheimer′s disease, Parkinson's disease and amyotrophic lateral sclerosis (ALS). The environmental chemicals shown to induce all these diseases include persistent organic pollutants (POPs), the plastic exudates bisphenol A and phthalates, low molecular weight hydrocarbons (LMWHCs) and polynuclear aromatic hydrocarbons (PAHs). It is reported here that though these chemicals differ widely in their chemical properties, reactivities and known points of attack in humans, a common link does exist between them. All are lipophilic species found in serum and they promote the sequential absorption of otherwise non-absorbed toxic hydrophilic species causing these diseases.

## Introduction

Neurological impairments (NIs), neurodevelopmental diseases (NDDs) and neurodegenerative diseases (NGDs) continue to increase dramatically worldwide. From 1990 to 2010, mental and behavioral disorders increased by more than 37%, Parkinson's disease increased by 75%, Alzheimer's disease doubled, autism increased by 30% and attention deficit hyperactivity disorder (ADHD) increased by 16% (Murray *et al.,* 2012; Vos, *et al.,* 2012). The increases in many epidemic and pandemic diseases, including neurological disorders, have been attributed to environmental exposures to exogenous toxic chemicals. The World Health Organization estimates that “as much as 24% of environmental disease is caused by environmental exposures that can be averted” (WHO, 2006).

Previously reported results have demonstrated the connection between lipophilic chemical exposure and type 2 diabetes (Zeliger, 2013b) and also with a cardiovascular disease (Zeliger, 2013a). It is reported here that such a connection also exists between lipophilic exposure and neurological impairments, neurodevelopmental diseases and neurodegenerative diseases.

It has been previously shown that mixtures of lipophilic and hydrophilic chemicals are toxic to humans at concentrations that are far below those known to be toxic for each of the components of such mixtures alone (Zeliger, 2003; Zeliger, 2011).

It has also been previously reported that exposures to the hydrophilic and lipophilic chemicals need not occur simultaneously but can occur sequentially, with the lipophilic substance exposure coming first and the hydrophilic exposure occurring some time later, provided that the lipophilic species is still retained in the body (Zeliger *et al.,* 2012). Such a sequential phenomenon has been demonstrated for the induction of type 2 diabetes (Zeliger, 2013b) and of a cardiovascular disease (Zeliger, 2013a). The case for such a mechanism for the induction of neurological disorders is proposed here.

The lipophiles associated with induction of neurological disorders can be long-lived persistent organic pollutants (POPs), which once absorbed can remain in the body's adipose tissue for up to 30 years or more and can be transferred to serum (Yu *et al.,* 2011). The lipophiles can also be intermediate-lived species, including polynuclear aromatic hydrocarbons (PAHs), bisphenol A (BPA) and phthalates, which can remain in the body for days or weeks (Stahlhut *et al.,* 2009; Kessler *et al.,* 2012; Li *et al.,* 2012). This applies also to low molecular weight hydrocarbons (LMWHCs), which are retained in body serum for days after absorption (Pan *et al.,* 1987; Zeliger *et al.,* 2012). The serum concentrations of the lower-lived species remain more or less in a steady state due to continuous exposure and absorption (Zeliger *et al.,* 2012), replacing the quantities lost via metabolism and elimination (Baselt, 2000).

Suggested mechanisms of neurotoxic action for some of these chemicals include oxidative stress, epigenetic effects and endocrine disruption (Kodavanti, 2005; Quaak *et al.,* 2013; Patri *et al.,* 2009; Lochhead *et al.,* 2010; Calderon-Garciduenas *et al.,* 2008; Colborn *et al.,* 1997; Patrick, 2009). Yet to date, no one mechanism can account for the neurological toxicity of this group of chemicals which differ widely in structure, chemical properties and reactivity. It is reported here, however, that there is indeed a unifying explanation for the induction of neurological diseases by this diverse group of chemicals. The studies referred to above show that accumulation of all of these chemicals in body serum was associated with increased incidence of neurological impairment, neurodevelopmental and neurodegenerative diseases. All these chemicals are lipophilic and all were shown to accumulate in body serum following exposure to them. Lipophilic chemicals were found to facilitate the absorption of hydrophilic chemicals across the body's lipophilic membranes (Zeliger, 2003; Zeliger, 2011). It is proposed here that the lipophilia of these exogenous chemicals induces neurological disorders by permeating lipophilic membranes, including the blood brain barrier, thus enabling the entry for toxic hydrophilic species that would otherwise not be absorbed.

## Methods

The results presented here are based upon the literature review of numerous studies, published both by this author and by others, on toxic effects of the chemicals involved, including case studies and epidemiologic studies. Adverse effects on health were in all instances diagnosed by appropriate clinical examinations and tests and chemical analytical data were generated in accordance with accepted protocols.

The total lipophilic load in serum is postulated as responsible for the induction of cardiovascular diseases (CVD). As used here, total lipophilic load refers to the total concentration of all exogenous lipophilic chemicals found in serum, without specification of individual chemical species.

## Results

As discussed below, exposures to POPs, plastic exudates, PAHs and LMWHCs have been found to be associated with neurological disorders. The POPs include polychlorinated biphenyls (PCBs), organochlorine pesticides (OCs), dioxins and furans and polybrominated diphenyl ethers (PBDEs). Plastic exudates include BPA and phthalates. LMWHCs include benzene, toluene, ethyl benzene, xylenes, C3–C8 aliphatics, gasoline, chlorinated methanes and ethanes and chlorinated ethylenes.

POPs, PAHs, LMWHCs, BPA and phthalates all have been shown to penetrate the blood-brain barrier (BBB) (Seelbach *et al.,* 2010; Qiu *et al.,* Escudar-Gilabert *et al.,* 2009; Gupta *et al.,* 1999; 2011; Hartz *et al.,* 2008; Caldreon-Garciduenas *et al.,* 2008; Sun *et al.,* 2002; Szychowski & Wojtowicz, 2013).

### I. Neurological impairments

NIs associated with lipophilic chemical exposure include central nervous system disorders (cognitive, motor and sensory), as well as peripheral nervous system maladies (neuropathies) (Gamble 2000; Baker 1988; Maruff *et al.,* 1988; Weintraub et al; 2000; Lee *et al.,* 2008b; Sendur *et al.,* 2009; Gupta *et al.,* 2011; Kodavanti, 2005; Patri *et al.,* 2009; Gamble, 2000; White & Proctor, 1997; Burbacher, 1993).

Recent research has shown that neurological impairment prevalence is increased by exposure to a number of different chemicals. These include persistent organic pollutants (POPs) – polychlorinated bipehenyls (PCBs) (Kodavanti 2005; Gascon *et al.,* 2013; Faroon *et al.,* 2001; Buters *et al.,* 2007; Fitzgerald *et al.,* 2012; Chia & Chu, 1984); organochlorine pesticides (OCs) (Jurewicz *et al.,* 2013b; Moses *et al.,* 2010; Iwaniuk *et al.,* 2006; Colosio *et al.,* 2003; Lee *et al.,* 2008b); dioxins and furans (Kodavanti 2005), and polybrominated biphenyl ethers (PBDEs) used as fire retardants (Kodavanti 2005; Buters *et al.,* 2007; Fitzgerald *et al.,* 2012; Widholm *et al.,* 2003; Thomke *et al.,* 1999; Michalek *et al.,* 2001; Sweeney *et al.,* 1993); BPA, widely used in the manufacture of plastic food containers and other applications (Viberg *et al.,* 2011; White *et al.,* 1997; Viberg *et al.,* 2012; Yolton 2011); phthalates, widely used as plasticizers for polyvinyl chloride (Jurewicz *et al.,* 2013a; Le Cann *et al.,* 2011; Yolton *et al.,* 2011), which are exuded from plastics; low molecular weight aliphatic and aromatic hydrocarbons (LMWHCs) and their chlorinated products which evaporate from gasoline, adhesives, paints and household products (Viaene, 2002; Maruff *et al.,* 1998; Burbacher, 1993; ATSDR, 2001; Lammers *et al.,* 2011); and polynuclear aromatic hydrocarbons (PAHs) which come from primary and secondary tobacco smoke inhalation and fuel combustion (ATSDR, 1995; He *et al.,* 2012; Patri *et al.,* 2009; Krivoshto *et al.,* 2008).

### II. Neurodevelopmental diseases

NDDs associated with lipophilic chemical exposure include autism spectrum disorders (ASD) (Winneke 2011; Roberts *et al.,* 2007; Roberts *et al.,* 2013; Larsson *et al.,* 2010; de Cock *et al.,* 2012; Cheslack-Postova *et al.,* 2013; Quaak *et al.,* 2013; Pessah *et al.,* 2008; Roberts and English 2007; McCanlies *et al.,* 2012; Volk *et al.,* 2011); attention deficit hyperactivity disorder (ADHD) (Polanska *et al.,* 2012; Pessah *et al.,* 2008; Roze *et al.,* 2009; Sagiv *et al.,* 2012; Sagiv *et al.,* 2010;de Cock *et al.,* 2012; Winneke 2011; Lee *et al.,* 2007; Harley *et al.,* 2013; Kim *et al.,* 2009; Quaak *et al.,* 2013); mental retardation (Grandjean & Landrigan, 2006); cerebral palsy (Grandjean & Landrigan, 2006), neural tube defects (Renn *et al.,* 2001; Brender *et al.,* 2010); hearing loss (Weitzman *et al.,* 2013); and other conditions (Eskenazi *et al.,* 2009); (Parron *et al.,* 2011; Loane *et al.,* 2013; Chen *et al.,* 2013; Steenland *et al.,* 2012; Wang, *et al.,* 2011; Dardiotis *et al.,* 2013;Caudle *et al.,* 2012;Weisskopf *et al.,* 2010;Moulton & Yang, 2012; Mayeux & Stern, 2012; Zaganas *et al.,* 2013; Sienko *et al.,* 1990; Vincenti *et al.,* 2012).

### III. Neurodegenerative diseases

NDGs associated with lipophilic exposure include Alzheimer's disease (Mates *et al.,* 2010; Chen *et al.,* 2013; Parron *et al.,* 2011; Steenland *et al.,* 2012; Zaganas *et al.,* 2013; Blanc-Lapierre *et al.,* 2012); Parkinson's disease (Caudle *et al.,* 2012; Wang *et al.,* 2011; Fleming *et al.,* 1994; Dardiotis *et al.,* 2013; Blank-Lapierre *et al.,* 2012); ALS (Vincenti *et al.,* 2012; Logroscino *et al.,* 2008; Uccelli *et al.,* 2007; Blanc-Lapierre *et al.,* 2012).

Table 1 summarizes the above findings associating NIs, NDDs and NDGs with the lipophilic chemicals identified as established or suspected causative agents of these diseases.

**Table 1:** Neurological disorders associated with lipophilic chemical exposures.

	POPS				PLASTIC EXUDATES		HYDROCARBONS	
	PCBs	OCs	PBDEs	Dioxins/Furans	phthalates	BPA	PAHs	LMWHCs
**NI**							
Cognitive effects	*	*	*	*	*	*	*	*
Motor deficits	*	*	*		*		*	*
Sensory deficits	*	*	*	*			*	*
Peripheral NS effects	*	*		*			*	*
**NDD**							
Autism	*	*	*		*	*	*	
ADHD	*	*	*	*	*	*	*	
**NDG**							
Alzheimer's disease		*		*	**	**	*	
Parkinson's disease	*	*	*	*	**	**	*	*
ALS		*		*				

* Established relationship

** Suspected relationship

## Discussion

The chemicals that are known to cause neurological diseases include POPs (PCBs, OCs, PBDEs, dioxins, furans, PFOEs), phthalates, BPA and hydrocarbons. These chemicals come from a variety of chemical classes that include chlorinated and brominated hydrocarbons, esters, ethers, polynuclear aromatic hydrocarbons, mononuclear aromatic hydrocarbons and straight chain aliphatic hydrocarbons. These chemicals differ widely in chemical properties, reactivity and rates of metabolism and elimination from the body.

The lipophiles associated with induction of neurological disorders can be long-lived POPs, which once absorbed can remain in the body's adipose tissue for up to 30 years or more and can be transferred to serum (Yu *et al.,* 2011). The lipophiles can also be intermediate-lived species, including PAHs, BPA and phthalates, which can remain in the body for days or weeks (Stahlhut *et al.,* 2009; Kessler *et al.,* 2012; Li *et al.,* 2012), as well as LMWHCs, which are retained in body serum for days after absorption (Pan *et al.,* 1987; Zeliger *et al.,* 2012). The serum concentrations of the lower-lived species remain more or less in a steady state due to continuous exposure (Zeliger, *et al.,* 2012) and absorption that replaces quantities lost via metabolism and elimination (Baselt, 2000).

Mechanisms by which environmental chemicals trigger neurological diseases have been proposed. These include: oxidative stress (Uttara *et al.,* 2009; Bolanos *et al.,* 2009), epigenetic effects (Jakovcevski & Akbarian, 2012; Urdinguio *et al.,* 2009) and endocrine distruption (Weiss, 2012; Mostafalou & Abdollahi, 2013; Colborn *et al.,* 1997). Compelling evidence has been presented to give validity to these mechanisms in some instances (see for example Urdinguio *et al.,* 2013). Until now, however, no single mechanism that accounts for the induction of a broad spectrum of neurological diseases has been proposed. The association with the onset of widely differing NIs, NDDs and NDGs with exposures to POPs, BPA, phthalates, PAHs and LMWHCs, chemicals which differ widely from each other, yet all of them are able to penetrate the blood-brain barrier, strongly suggests a lipophile-dependent mechanism for the induction of CVDs. The concept of lipophilic chemicals serving to assist the penetration of hydrophilic therapeutic drugs through the blood-brain barrier is well established (Pardridge, 2012; Patel *et al.,* 2009; Filmore, 2002; Seelig *et al.,* 1994), lending credence to the mechanism suggested here.

It has been previously shown that mixtures of toxic chemicals containing at least one lipophilic and one hydrophilic agent produce effects not predictable from the known toxicology of the individual species. These effects include attack on organs and systems not known to be impacted by the individual species, low-level toxicity induced by exposures to concentrations far below those known to be toxic by single chemicals in the mixtures and enhanced toxicity to humans (Zeliger, 2003). The correlation presented here between lipophilic absorption with sequential hydrophilic absorption corroborates well these findings. In all the published studies, the levels of lipophiles in blood are far lower than those known to be acutely toxic for the individual species.

POPs are long-lived and accumulate in white adipose tissue (WAT) from which they can pass to the blood and be transported around the body (Yu *et al.,* 2011; Mullerova & Kopecky, 2007; Covaci *et al.,* 2002). Due to the slow rates of metabolism and elimination, once absorbed, POPs can persist in the body for 30 years or longer and can build up with time to toxic concentrations (Yu *et al.,* 2011; Gallo *et al.,* 2011). This bioaccumulation of POPs with time over many years accounts for the delayed onset of disease following initial exposure.

The lower molecular weight of NI, NDD and NDG inducing chemicals (phthalates, BPA, PAHs and LMWHCs), absorbed even at toxic concentrations, are more rapidly metabolized/eliminated. Nevertheless, they persist in body serum for days to weeks (Stahlhut *et al.,* 2009; Koch *et al.,* 2004; Li *et al.,* 2012; Pan *et al.,* 1987). Accordingly, short-term toxic concentrations from single exposures to these are fairly rapidly reduced. All of these chemicals, however, are ubiquitous in the environment as air, water or food contaminants, resulting in fairly continuous absorption and maintenance of steady-state concentrations in the blood of those who are continually exposed. Such a scenario applies as well to those who take some pharmaceutics on a regular basis and produce fairly constant levels of lipophiles in the blood stream (Zeliger *et al.,* 2012; and Culver *et al.,* 2012).

The chemicals described above have one characteristic in common, they are all lipophiles. Although the exposure levels of these lipophilic species are much lower than their known toxic levels, they are high enough to provide a vehicle for the sequential absorption of toxic hydrophilic species (Zeliger *et al.,* 2012; Zeliger, 2013b). It is well known that mixtures of lipophilic and hydrophilic species induce low-level toxic effects and unanticipated points of attack (Zeliger, 2003; Zeliger 2011). It is proposed here that combinations of low-level lipophilic/hydrophilic mixtures act as agents for neurological disease induction.

We suggest that the structure of the lipophile is not the critical point. Rather, it is the lipophilicity and total serum load of a lipophilic species that is the determining factor in triggering neurological disease. Once a steady-state critical dose of a lipophile is reached, the body is ripe for sequential attack by a hydrophilic species, with the mixture of a lipophilic and a hydrophilic species capable to attack even at low levels of exposure (Zeliger, 2003; Zeliger, 2011, Zeliger *et al.,* 2012).

Support for this proposal comes from a consideration of other environmental diseases that have been attributed to exposures to these chemicals. Exposures to POPs, hydrocarbons and plastic exudates have been associated with metabolic diseases including type 2 diabetes, metabolic syndrome and obesity (Zeliger, 2013b; Lee *et al.,* 2010; Carpenter 2008). Exposures to the lipophilic chemicals discussed above have also been associated with a broad spectrum of cardiovascular diseases (Humblet *et al.,* 2008). These include myocardial infarction (Mustafic *et al.,* 2012, Wichmann *et al.,* 2013); atherosclerosis (Whayne, 2011, Lind *et al.,* 2012); hypertension (La Merrill *et al.,* 2013; Sergeev & Carpenter, 2011; Lind & Lind, 2012; Ha *et al.,* 2009; Valera *et al.,* 2013), coronary heart disease (Shankar *et al.,* 2012, Lind and Lind 2012), peripheral heart disease (Shankar *et al.,* 2012; Lind & Lind, 2012); ischemic heart disease (Toren *et al.,* 2007; Costello *et al.,* 2013; Burstyn *et al.,* 2005); and impact on cardiac autonomic function (Wu *et al.,* 2012).

Other diseases associated with exposure to lipophilic chemicals include: immunological disorders (Hertz-Picciotto *et al.,* 2008; Noakes *et al.,* 2006; Tryphonas, 1998), musculoskeletal disorders (Lee *et al.,* 2007), reproductive interferences (EPA, 2008; Nishijo *et al.,* 2008; Herz-Picciotto *et al.,* 2008), endocrine disruption (Snyder & Mulder, 2001; Colborn *et al.,* 1997), autoimmune diseases (Koch *et al.,* 2013; Sozeri *et al.,* 2012; Farhat *et al.,* 2011; Gregory *et al.,* 2008; Dahlgren *et al.,* 2007;) and periodontal disease (Lee *et al.,* 2008a).

The onset of many different cancers has also been associated with exposures to the chemicals described here. A discussion of environmental causes of cancer, however, is beyond the scope of this presentation. Zeliger 2004 and Zeliger 2011 offer an introduction to this subject.

People are routinely exposed to many other lipophilic chemicals that are retained in body serum. These include mycotoxins produced by mold and found in wet environments and in comtaminated food (Brasel *et al.,* Peraica *et al.,* 1999; 2004; Brasel *et al.,* 2004; Reddy & Bhoola, 2010; Bennett & Klich, 2003; Brewer *et al.,* 2013), anti-oxidants and other preservatives added to foods and cosmetics, including triclosan, an antibacterial compound widely used in tooth paste, cleaners and other consumer products (Queckenberg *et al.,* 2010; Sandborgh-Englund *et al.,* 2006), butylated hydroxyanisole (BHA) and butylated hydroxytoluene (BHT) (Surak *et al.,* 1977; Verhagen *et al.,* 1989; Conning & Phillips, 1986). Other compounds are chlorinated derivatives of methane that are byproducts (DBPs) of the disinfection of water by chlorine, including chloroform and the bromo-chloro-methanes (Zeliger, 2011), the chlorinated derivatives of ethane, including 1,1,1-trichloroethane, trichloroethylene, tetrachloroethylene that arise from cleaning products and contamination of aquifers (Zeliger, 2011), pharmaceuticals that are found in contaminated drinking water in many cities (Donn, 2008); antioxidants put into foods and cosmetics for preservation purposes, including BHA and BHT (Conning & Phillips, 1986; Verhangen *et al.,* 1989), brominated vegetable oil, used to stabilize citrus-flavored soft drinks (Bernal *et al.,* 1986; Bendig *et al.,* 2013) and lipophilic pharmaceuticals, examples of which are statins, taken regularly (Culver *et al.,* 2012; Zeliger *et al.,* 2012).

The prevalence of neurological diseases discussed here as well as the other diseases cited have all increased dramatically in the past half century. For example, the prevalence of the bipolar disorder and of schizophrenia has increased in the range of 40% from 1990 to 2010 (Murray *et al.,* 2012), the prevalence of Alzheimer's disease is expected to double every 20 years (Mayeux & Stern, 2012). Such an increase can only be explained by environmental consideration and it corresponds to the worldwide increased use of POPs, plastic additives and other chemicals, fossil fuel and the environmental pollution associated with their use and discharge (EIA, 2013; Chen & McCarl, 2001; Colborn *et al.,* 1997).

As previously discussed, PAHs emanating from the combustion of fossil fuels and tobacco are considered to induce many environmental diseases. Several of the studies cited have made the association with inhalation of fine particles rather than with the PAHs (Costello *et al.,* 2013; Toren *et al.,* 2007). It has been shown, however, that the toxicity of the particles is due to the adsorption of the PAHs on the solid particles and the subsequent partitioning from such particles onto and through lipophilic membranes (Yokota *et al.,* 2008). The fine particles serve as vehicles to deliver the PAHs deep into the lungs, where these compounds are absorbed.

As seen in Table 1, not all of the lipophiles identified here have been associated with all of the diseases noted. This is due to the fact that causative studies are yet to be carried out for many chemical species.

It is to be noted that although the literature relating neurological disease to other exogenous lipophilic chemicals is scanty, mycotoxin exposures have been associated with neurological disease (Pestka *et al.,* 2008; Doi & Uetsuka, 2011; Moldes-Anaya *et al.,* 2012). Mycotoxins, as well as the widely used lipophilic disinfectant triclosan have been shown to accumulate in serum (Brewer *et al.,* 2013; Queckenberg *et al.,* 2010; Sandborgh-Englund *et al.,* 2006) and as such, they contribute to the total lipophilic load.

## Conclusion

The prevalence of neurological diseases, including NIs, NDDs and NDGDs is increasing rapidly throughout the world. The evidence presented here strongly suggests that this increase is due in large part to increased exposure to exogenous lipophilic chemicals which, though varying widely in structure, toxicology, chemical reactivity and retention time in the body, render the body susceptible to attack via subsequent exposure to low levels of hydrophilic toxins that would otherwise not be absorbed. The lipophilic chemicals can be POPs that are metabolized and eliminated slowly, or BPA, phthalates, PAHs, LMWHCs and other lipophilic species that are eliminated from the body more rapidly, but are constantly replenished in the body from polluted air and water and contaminated food. The accumulation of lipophilic chemicals in the body proceeds until a critical lipophilic load level is reached, at which point the body is vulnerable to attack by low levels of toxic hydrophilic chemicals that would otherwise not be toxic. Sequential absorption of lipophiles followed by hydrophiles provides a unified explanation of how low levels of rather different environmental pollutants are responsible for the alarming increase of neurological diseases.
